# The Efficiency of Taurolidine Lock Solution in Preventing Catheter-Related Bloodstream Infections in Children with Intestinal Failure

**DOI:** 10.3390/medicina61122188

**Published:** 2025-12-10

**Authors:** Betül Aksoy, Şenay Onbaşı Karabağ, Yeliz Çağan Appak, Selen Güler, Sinem Kahveci, Dilek Yılmaz, Maşallah Baran

**Affiliations:** 1 Department of Pediatric Gastroenterology, Hepatology and Nutrition, Faculty of Medicine, Izmir Katip Celebi University, İzmir City Hospital, 35540 Izmir, Türkiye; senayonbasikarabag@gmail.com (Ş.O.K.); yelizcagan@yahoo.com (Y.Ç.A.); selenguler6@hotmail.com (S.G.); dr_skahveci@hotmail.com (S.K.); mbaran2509@gmail.com (M.B.); 2 Department of Pediatric Infectious Disease, Faculty of Medicine, Izmir Katip Celebi University, İzmir City Hospital, 35540 Izmir, Türkiye; drdilekyilmaz@hotmail.com

**Keywords:** pediatrics, intestinal failure, central venous catheters, taurolidine–citrate, catheter-related bloodstream infection

## Abstract

*Background and Objectives:* Catheter-related bloodstream infections (CRBSIs) are one of the most severe complications in children with intestinal failure (IF) who require long-term parenteral nutrition (PN). Taurolidine–citrate solution (TCS), with proven antimicrobial and antibiofilm properties, has been proposed as a promising alternative to heparin locks for preventing infection. The aim is to evaluate the efficacy and safety of the TCS in reducing the rates of CRBSI and pathogen-specific infections in pediatric patients with indwelling central venous catheters (CVCs) who are receiving PN. *Materials and Methods:* This retrospective study included 48 pediatric IF patients treated at an intestinal rehabilitation and transplantation center in Türkiye. Patients received either TCS or heparinized saline (0.9% saline solution containing 100 IU of heparin) as a catheter lock. Infection data were extracted from medical records and expressed as events per 1000 catheter days. Group comparisons were performed using non-parametric tests, and Poisson regression was applied to calculate rate ratios (RRs) and 95% confidence intervals (CIs). Adjusted rate ratios were obtained from a Poisson regression model that included the following variables: age, sex, diagnosis category, ostomy status, catheter type, and follow-up duration. Log(catheter-days) was incorporated as an offset term. Overdispersion was assessed and not detected. *Results:* The crude CRBSI rate was lower in the TCS group than in the heparinized saline group (29.4 vs. 42.8 per 1000 catheter days), though this difference was not statistically significant (*p* = 0.383). However, after adjustment by Poisson regression, TCS use was significantly associated with reduced infection rates (adjusted RR = 0.78, 95% CI = 0.70–0.87, *p* < 0.001). TCS use was also significantly associated with reduced rates of Gram-positive (RR = 0.78, *p* = 0.006), Gram-negative (RR = 0.48, *p* < 0.001) and fungal (RR = 0.63, *p* < 0.001) infections. No adverse events were observed among the TCS group. *Conclusions:* Standardized TCS lock therapy effectively and safely reduces CRBSIs in pediatric patients with IF, particularly those caused by Gram-negative and fungal organisms. These results support the use of TCS as a prophylactic option for preventing infection in long-term CVC use.

## 1. Introduction

Intestinal failure (IF) is defined as the inability of the intestine to absorb enough fluids and nutrients via parenteral nutrition (PN) to sustain life and promote growth in children. It results from the anatomical or functional loss of a significant proportion of the intestine. The current prevalence of IF in children is estimated to be between 9.6 and 16.6 per million, and this figure appears to be increasing [1,2,3]. PN therapy is associated with potentially life-threatening complications, such as infection and liver disease. Intestinal rehabilitation involves implementing medical and surgical strategies to progressively restore intestinal function, with the ultimate aim of weaning patients off PN. Intestinal transplantation (IT) is an option for patients with irreversible IF.

Central venous catheters (CVCs) should be used for PN-dependent children with IF. Catheter-related bloodstream infections (CRBSIs) are among the most common and serious complications of CVC use. The incidence rate of CRBSIs exceeds 10 per 1000 catheter days in pediatric populations, substantially contributing to increased mortality, overall treatment costs, and a further increased risk of infection with long-term catheter use [4,5,6]. The most frequent cause of CRBSIs is catheter hub contamination during handling by healthcare professionals, leading to endoluminal colonization and infection. Due to an increased risk of bacterial and fungal translocation across a compromised intestinal mucosal barrier, children with intestinal failure may exhibit an elevated susceptibility to bloodstream infections. Consistent with this pathophysiology, a broad spectrum of organisms, including enteric and oral flora as well as yeast species, has been implicated in CRBSIs in this population [7]. Various strategies have been implemented to reduce CRBSI rates, including improvements in infection control and maintenance and insertion bundles. However, heparin, which is routinely used to lock the catheter and prevent clotting, is thought to promote bacterial growth inside the catheter hub [8]. While prophylactic antimicrobial lock solutions are beneficial in preventing CRBSIs, they also increase the risk of selecting resistant microorganisms [9,10]. Taurolidine [bis-(1,1-dioxoperhydro-1,2,4-thiadiazinyl-4)- methane] is a naturally occurring derivative of taurine that hydrolyzes into two antimicrobial parts: formaldehyde and methylene glycol. These bind to the walls of bacteria and fungi, killing these organisms [11]. Taurolidine also prevents CRBSI caused by Gram-positive and Gram-negative bacteria, such as methicillin-resistant Staphylococcus aureus, coagulase-negative staphylococci and vancomycin-resistant enterococci [12].

The present study aimed to evaluate the efficacy and safety of using a taurolidine and citrate solution (TCS) to reduce the incidence of CRBSI in children with IF receiving long-term PN. It was hypothesized that TCS would be significantly more effective than 0.9% saline and heparin locks at reducing infection rates.

## 2. Materials and Methods

### 2.1. Study Design and Population

This retrospective study was conducted at the Unit of Intestinal Rehabilitation and Transplantation Center in İzmir Tepecik Research Hospital and İzmir City Hospital, Türkiye. A retrospective study design was chosen due to the low prevalence of pediatric IF and the challenges associated with conducting randomized prospective trials in this rare population. This design allowed for the inclusion of real data reflecting routine clinical practice in a tertiary intestinal rehabilitation setting.

Patients under the age of 18 years with IF of any etiology who received PN via a CVC were included. Patients with immunodeficiency or comorbidities that predispose them to infection and with incomplete medical records were excluded from the study.

The patients were divided into two groups according to the locking solution used for catheter closure. In the first group, 1 mL of Taurolock^TM^ (TauroPharm GmbH, August-Bebel-Straße 51, D-97297 Waldbüttelbrunn, Germany) was administered into the catheter lumen every day and stayed in the catheter lumen for 2 h. TauroLock™ comprises a 4% TCS. The citrate component prevents blood coagulation and platelet aggregation, thereby reducing the likelihood of catheter occlusion. In the second group, closure treatment was applied by leaving it in the catheter lumen for two hours each day with heparinized saline containing 1 mL of 0.9% saline and 100 IU of heparin. The choice of locking solution depended on the institutional protocol in use at the time of catheter insertion. Catheter care procedures were performed using aseptic techniques in accordance with the standardized institutional protocol. Because the transition from heparinized saline to taurolidine-citrate lock solutions occurred gradually during the study period, the choice of lock solution depended partly on when the catheter was inserted. Consequently, the two study groups were not fully contemporaneous, which is an inherent feature of the retrospective design and should be considered when interpreting comparative infection rates. Although a formal sensitivity analysis could not be conducted, we documented the temporal distribution of lock solution use to provide additional context. Heparinized saline was predominantly used between 2011 and 2017, while taurolidine-citrate became the standard lock solution starting in 2018. A descriptive review of annual infection-control reports showed no major center-wide trends in CRBSI incidence across these years.

The dwell time of the locking solution was standardized at approximately two hours per day, in line with the institutional protocol. Although longer dwell times ranging from 2 to 24 h have been reported in the literature, shorter durations were adopted in our center to minimize catheter downtime and ensure daily clinical monitoring. This dwell time was applied consistently to all patients to ensure procedural uniformity.

Medical records were reviewed to obtain demographic, clinical, catheter-related and infectious data. The following were recorded: demographic characteristics; follow-up duration; underlying diagnosis; presence of an ostomy; history of IT use of home PN; weaning off PN; adverse events; and intestinal failure-associated liver disease (IFALD), that is, liver injury due to IF and PN in the absence of any primary parenchymal liver disease. Catheter-related parameters such as catheter type, duration of cumulative catheter days and number of catheter removals were also noted.

### 2.2. Definition of CRBSI

The CRBSI results of patients were collected using retrospective data. The diagnosis of CRBSI was based on the clinical criteria recommended by the Center for Disease Control and the São Paulo State surveillance manual [13]. Only episodes with at least one clinical sign (fever, chills, or hypotension) and a positive blood culture for an identifiable pathogen without an alternative source of infection were included. Therefore, culture-negative or clinically suspected cases were not considered CRBSIs and were excluded from the study. Additionally, polymicrobial episodes were not included in the study. An episode was considered a new infection if a new organism was grown in a culture after a previous culture or if the culture was positive at least two weeks after completing an antibiotic course. The incidence rate of CRBSI was calculated as the number of infection episodes divided by the total number of catheter days, and expressed per 1000 catheter days [14]. The organisms in blood cultures were classified as Gram-positive, Gram-negative or fungal and counted according to CRBSI episode. The incidence rate of CRBSI was compared between two groups.

### 2.3. Ethical Considerations

The study was conducted in accordance with the Declaration of Helsinki and approved by the local Scientific-Ethical Committee (approval no. 2021/11-27).

### 2.4. Statistical Analysis

Categorical variables (sex, diagnosis classification, presence of ostomy, IT, IFALD, home PN, weaning off PN, type of CVC, and type of causative organism) were analyzed using the chi-square (χ^2^) test and are expressed as numbers and percentages. The Kolmogorov–Smirnov and Shapiro–Wilk tests were used to assess the normality of continuous variables. Non-normally distributed continuous variables (age, follow-up period, cumulative catheter duration, CVC removals, CRBSI rates, and organism-specific CRBSI rates) were compared between groups using the Mann–Whitney U test. Poisson regression models were used to estimate rate ratios (RRs) and 95% confidence intervals (CIs) for overall and organism-specific CRBSI rates. The adjusted models included the following prespecified covariates: age, sex, diagnosis category, presence of an ostomy, catheter type, and follow-up duration. The log of catheter days was incorporated as an offset term to account for differing exposure times. During model building, variables such as intestinal transplantation, home parenteral nutrition, and cumulative catheter exposure were evaluated. However, due to their limited frequency, they resulted in model instability and were not included in the final adjusted model. Overdispersion was assessed using the Pearson chi-square/df statistic and was not detected; therefore, standard Poisson models were retained. Non-parametric tests (Mann–Whitney U and chi-squared) were applied because most continuous variables were not normally distributed, and the results are presented as median and interquartile range (IQR) values. All statistical analyses were performed using the Statistical Package for the Social Sciences software (version 25.0; IBM Corp., Armonk, NY, USA). A *p*-value < 0.05 was considered statistically significant.

## 3. Results

### 3.1. Patient Characteristics

A total of 48 patients with IF were included in the study. The median age at baseline was 7.7 months (range 0.4–384 months), and 54.2% of patients were female. The median follow-up duration was 7.2 months (range 1–102 months). The underlying diagnoses were short bowel syndrome (56.3%), followed by intestinal dysmotility (25.0%) and congenital diarrhea (18.7%). Of the patients, 60.4% had an ostomy, 20.8% underwent IT, and 8.3% had IFALD.

TCS was administered to 25 patients, while 23 patients received heparinized saline. Baseline demographic and clinical characteristics were similar between the two groups (Table 1). There were no significant differences in the distribution of sex, diagnosis, presence of ostomy and follow-up duration between the groups (*p* > 0.05). Due to the low frequency of certain variables, such as intestinal transplantation, IFALD, home PN, and PN weaning, no statistical comparisons were performed.

### 3.2. Catheter Characteristics and Outcomes

The median cumulative catheter duration for all patients was 135 days (range 11–3060 days), with no significant difference found between the TCS and heparinized saline groups (*p* = 0.960). The mean rate of catheter removal for any reason was 27.7 ± 21.3 per 1000 catheter days, with comparable rates observed in both groups (*p* = 0.305). In terms of catheter type, 31.3% of cases involved nontunneled CVC, while implantable ports accounted for 35.4%. There was no significant difference between the groups in this respect (*p* = 0.137).

### 3.3. Catheter-Related Bloodstream Infections

The overall CRBSI rate was 35.3 ± 32.8 per 1000 catheter days. Although the mean CRBSI rate was lower in the TCS group (29.4 ± 23.4 per 1000 catheter days) than in the heparinized saline group (42.8 ± 41.0 per 1000 catheter days), this difference was not statistically significant (*p* = 0.383) (Table 2).

**Figure 1:**
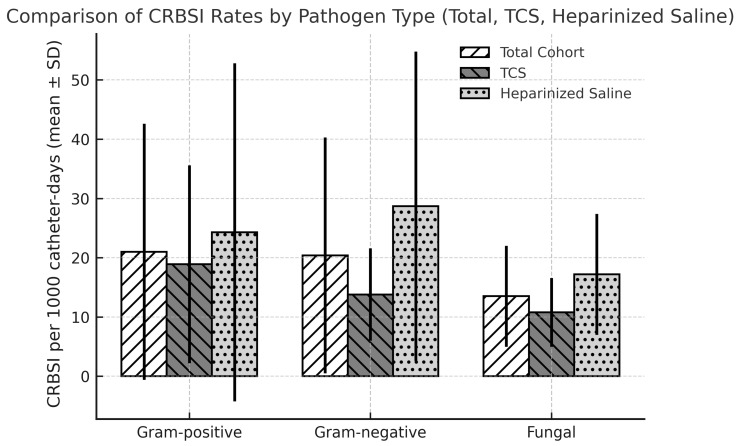
Comparison of catheter-related bloodstream infection (CRBSI) rates per 1000 catheter days among the total cohort, the TCS group and the heparinized saline group, categorized by pathogen type. Bars represent mean ± SD.

Analysis of the organisms responsible for the infections revealed that 47.9% were caused by Gram-positive bacteria, 58.3% by Gram-negative bacteria and 69.4% by fungal species. There were no significant differences between the groups (*p* > 0.05). However, when the incidence rate of infection per 1000 catheter days was calculated by pathogen type, Gram-negative and fungal infections were significantly more prevalent in the heparinized saline group than in the TCS group (*p* = 0.022 and *p* = 0.037, respectively) (Figure 1).

A Poisson regression analysis was conducted to evaluate the impact of TCS usage on infection rates. The results showed that TCS use was linked to significantly lower infection rates for all measured outcomes. Specifically, compared to those in the heparinized saline group, patients in the TCS group exhibited reductions in infection rates of 22% for CRBSI, 37% for fungal infections, 52% for Gram-negative infections and 22% for Gram-positive infections. All reductions were statistically significant (*p* < 0.01) (Table 3).

### 3.4. Adverse Events

None of the patients experienced any adverse effects, hypersensitivity reactions, hematological side effects or organ toxicity that could potentially be associated with the use of TCS.

## 4. Discussion

This retrospective study of pediatric patients with IF who were dependent on PN showed that the use of TCS was associated with a statistically significant reduction in infection rates compared to heparin containing 0.9% saline locks for all measured outcomes. Poisson regression analysis revealed reductions of 22%, 52% and 22% in overall CRBSIs, Gram-negative infections and Gram-positive infections, respectively. These findings are consistent with the recent Spanish Society of Pediatric Gastroenterology, Hepatology and Nutrition practical guidelines, which recommend taurolidine as the preferred prophylactic lock solution for pediatric patients with IF on PN due to its proven efficacy in preventing catheter-related infections [15]. Our study is one of the few evaluations of TCS lock therapy in children with IF, who are a clinically vulnerable and understudied population. In addition, a standardized, reproducible lock protocol was evaluated, with the focus being solely on pediatric patients in this research. Importantly, our analysis revealed an association between TCS use and lower rates of CRBSIs caused by specific pathogens, particularly Gram-negative and fungal organisms. This effect has not been consistently reported in previous pediatric studies.

Our findings corroborate and extend the existing evidence supporting the antimicrobial efficacy of TCS in pediatric patients with IF and long-term CVC. A meta-analysis by Sun et al. reported a significant reduction in CRBSIs with taurolidine use in pediatric patients, with a pooled risk ratio of 0.23 (95% CI 0.10–0.53) compared with heparin locks [14]. Similarly, in a randomized controlled trial, Tribler et al. demonstrated that a taurolidine–citrate–heparin lock completely prevented CRBSI (0 events), as opposed to seven episodes (1.0/1000 catheter days) in the heparin group [16]. In a multicenter study, Wouters et al. observed an approximately 80% reduction in the risk of infection in new catheter users receiving taurolidine, further highlighting its preventive benefits for patients receiving home PN [17]. Similar results were reported by Chong et al., who evaluated the use of a taurolidine–citrate lock solution in pediatric hematology–oncology and gastrointestinal failure patients, who had high baseline rates of CRBSIs [18]. In the open-label study, the mean CRBSI rate decreased from 14.44 to 2.45 episodes per 1000 catheter days (rate ratio 0.20, *p* < 0.001), representing an 80% overall reduction. Although the risk reduction observed in our study was smaller than that reported in some large-scale randomized trials, this can be attributed to differences in baseline CRBSI rates, study design and patient characteristics. Additionally, Nerstrøm et al. conducted a randomized, double-blind, placebo-controlled trial with adult patients receiving home parenteral support for chronic IF. They demonstrated that a 1.35% taurolidine lock reduced recurrent CRBSIs by 77% compared to saline and decreased catheter removals due to infection by 91% [19]. These results support the findings of the present study that taurolidine is effective not only in primary prevention but also in reducing the recurrence of infections in high-risk populations.

Building on these studies, our findings demonstrate that TCS is not only effective at reducing bacterial and fungal CRBSIs in a pediatric IF cohort, but also more effective than heparinized saline. This is particularly important given that this group is particularly susceptible to infectious morbidity. Taurolidine hydrolyzes to produce derivatives containing methylol that bind to components of the microbial cell wall, causing irreversible structural damage and preventing colonization. Importantly, taurolidine’s mechanism of action is independent of traditional antibiotic pathways, thus minimizing the risk of microbial resistance—a crucial advantage for patients requiring chronic catheterization [15,20]. Similarly, Alfieri et al. published a comprehensive scoping review encompassing over 300 clinical studies, confirming the broad antimicrobial and antifungal efficacy of taurolidine in both pediatric and adult populations. The review also emphasized taurolidine’s excellent safety profile, negligible propensity for resistance development and ability to prevent microbial colonization and biofilm formation on catheter surfaces [21].

In addition to lacking intrinsic antimicrobial activity, traditional heparin locks have several drawbacks that may adversely affect catheter safety in pediatric patients with intestinal failure. Experimental studies have demonstrated that heparin can stimulate Staphylococcus aureus biofilm formation and promote bacterial adherence to catheter surfaces. This increases the likelihood of persistent or recurrent catheter-related infections [8]. Furthermore, randomized clinical data indicate that, compared with taurolidine-containing lock solutions, heparin-based locking regimens do not reduce infection risk and may permit ongoing microbial colonization [16]. Collectively, these limitations reinforce the rationale for considering taurolidine–citrate as a non-antibiotic alternative with more favorable microbiological properties.

The statistically significant protective effect observed across all microbial categories (Gram-positive, Gram-negative and fungal) in our study further highlights the broad-spectrum antimicrobial properties of taurolidine. This broad effect has also been highlighted in meta-analyses of adult and pediatric cohorts. In these studies, taurolidine was found to reduce the risk of catheter-related bloodstream infection by around 70%, when compared with heparin or saline locks [22]. A systematic review and meta-analysis of taurolidine lock solutions (based on six randomized controlled trials involving 431 patients and 86,078 catheter days) revealed a notable decrease in the occurrence of CRBSIs when compared with heparin lock solutions [20]. Taurolidine has only a few reported side effects and continued use is not associated with bacterial resistance development [10].

The prevalence of fungal and Gram-negative organisms in our cohort was higher than in other pediatric intestinal failure populations [7]. This difference may reflect center-specific epidemiology due to variations in baseline patient complexity, antimicrobial exposure patterns, catheter length, length of stay, and local microbiological ecology. When interpreting pathogen distribution, these contextual factors should be considered, as they may limit the generalizability of organism-specific rates.

The most significant benefit observed in our study was for Gram-negative infections, which were reduced by over half among the TCS group (RR = 0.48). This finding is consistent with in vitro data indicating that TCS disrupts the integrity of the lipopolysaccharide layer in Gram-negative bacteria and inhibits the formation of biofilms on catheter surfaces [12]. Furthermore, the reduction in Candida-associated infections is consistent with reports of TCS’s antifungal activity, which is likely mediated by aldehyde derivatives that damage fungal cell walls [10]. Additionally, infection rates are influenced by various interrelated factors, such as the severity of the underlying disease, the type of catheter used and the quality of home care practices. In the present study, the groups had similar baseline characteristics, including sex, age, diagnosis, follow-up duration, cumulative catheter exposure, and type of CVC.

In terms of safety, no taurolidine-related adverse events, hypersensitivity reactions, hematological abnormalities or organ toxicities were observed in our cohort, which confirms its excellent tolerability. Although a case of taurolidine-induced anaphylaxis in a pediatric patient has been reported, our experience supports the favorable safety profile of the compound in IF patients [23]. In other long-term catheterized populations, such as hemodialysis patients, taurolidine–heparin lock solutions have also been shown to be effective without compromising catheter patency or systemic safety [11].

Our findings should be interpreted with caution, particularly with regard to the lock dwell time. Most of our patients required total PN, so a standardized two-hour dwell time was used. This differs from the longer dwell durations (ranging from several hours to 24 h) commonly reported in clinical practice and in prior studies of taurolidine lock therapy [16,17]. These procedural differences may influence antimicrobial exposure within the catheter lumen and could account for some of the variations in reported effectiveness across institutions.

Although the reduction in infection rates observed in this study was both clinically and statistically significant, there were a few limitations: the retrospective design, relatively small sample size, and limited follow-up period may restrict the generalizability of our findings. Furthermore, although catheter removal rates were comparable between the TCS and heparinized saline groups, the study did not assess hospitalization duration or the health economic impact—parameters which have shown favorable trends with taurolidine use in previous studies [24,25]. However, insufficient data prevented an evaluation of catheter removal rates due to infection alone. Another limitation is the gradual institutional transition from heparinized saline to TCS over time, where the choice of lock solution may partially reflect the period of catheter insertion. Therefore, the observed differences may partially reflect improvements in clinical protocols rather than the lock solution alone. Additionally, adjustments for year and center were not possible given the data limitations. Sensitivity analyses restricted to overlapping periods when both solutions were in use could not be conducted. Moreover, clustering by patient could not be modeled. This was due to the small number of repeated CRBSI episodes per individual. These gaps highlight the need for future prospective studies designed to evaluate infection outcomes and broader clinical and economic endpoints.

Despite these limitations, the consistent trend of reduced CRBSI rates across all microbial categories strengthens the clinical importance of our findings and suggests that the use of TCS is associated with lower CRBSI rates and appears to be well tolerated. However, due to the retrospective design of the study, causal inferences cannot be made.

### Future Perspectives

Future multicenter, prospective, randomized studies focusing on pediatric IF are essential to validate the observed associations and refine the clinical use of taurolidine–citrate lock solutions. Due to the significant variability in dwell times, concentrations, and lock frequencies reported across centers, there is an urgent need for standardized, evidence-based protocols to ensure consistent application and enhance inter-institutional comparability. Such standardized approaches would also facilitate harmonized data collection and strengthen future meta-analytic efforts. Furthermore, future research should expand beyond infection-related endpoints to include long-term cost-effectiveness, catheter longevity, hospital burden, and patient and caregiver quality of life. Integrating these broader clinical and economic dimensions is critical to determining the overall value of taurolidine lock therapy and guiding its optimal implementation in intestinal rehabilitation programs.

## 5. Conclusions

This retrospective, non-randomized cohort study found that the use of a TCS was associated with lower rates of catheter-related bloodstream infections, including Gram-negative and fungal episodes, in children with IF who received PN. These results imply a potential benefit of taurolidine–citrate for routine catheter care. However, due to the historical control study design, causal inferences cannot be made. Prospective, randomized studies are needed to confirm these associations and define standardized protocols regarding optimal dwell time, concentration, and frequency.

## Figures and Tables

**Table 1:** Baseline demographic and clinical characteristics of the study population.

	Total	TCS	Heparinized Saline	*p*
**Patient characteristics,** n (%)	48 (100)	25 (52.0)	23 (48.0)	-
**Sex (Female),** n (%)	26 (54.2)	16 (64.0)	10 (43.5)	0.246 ^a^
**Age at baseline,** months median (IQR)	7.7 (4–17.5)	8.5 (5–16)	6 (2.7–20)	0.183 ^b^
**Follow-up period,** months median (IQR)	7.2 (2.6–14)	7 (2–14)	7.5 (3–18)	0.810 ^b^
**Diagnosis classification,** n (%)				0.138 ^a^
Short bowel syndrome	27 (56.3)	11 (44.0)	16 (69.6)	
Intestinal dysmotility	12 (25.0)	7 (28.0)	5 (21.7)	
Congenital diarrhea	9 (18.7)	7 (28.0)	2 (8.7)	
**Patients with an ostomy,** n (%)	29 (60.4)	12 (48.0)	13 (56.5)	0.083 ^a^
**Intestinal transplantation,** n (%)	10 (20.8)	2 (8.0)	8 (34.7)	-
**IFALD,** n (%)	4 (8.3)	1 (4.0)	3 (13.0)	-
**Home PN,** n (%)	4 (8.3)	3 (12.0)	1 (4.3)	-
**Weaning off PN,** n (%)	11(22.9)	4 (16.0)	7 (30.4)	-

TCS = taurolidine–citrate; IFALD = intestinal failure-associated liver disease; PN = parenteral nutrition. ^a^. Pearson’s exact chi-square test; ^b^. Mann–Whitney U test.

**Table 2:** Catheter characteristics and CRBSI outcomes.

	Total	TCS	Heparinized Saline	*p*
**Cumulative Catheter** **Duration, days** median (IQR)	135 (60–300)	90 (60–330)	165 (60–412.5)	0.960 ^b^
**CVC removals due to any reason/1000 catheter days,** median (IQR)	13.3 (8.3–33.3)	13.6 (7.9–33.3)	12.1 (8.7–33.3)	0.305 ^b^
**Type of CVC,** n (%)				0.137 ^a^
Nontunneled CVC	15 (31.3)	11 (44)	4 (17.4)	
Implantable port	17 (35.4)	7 (28)	10 (43.5)	
Both	16 (33.3)	7 (28)	9 (39.1)	
**CRBSI/1000 catheter Days,** median (IQR)	33.3 (8.6–53.1)	33.3 (11.1–39.3)	36.3 (3.0–83.3)	0.383 ^b^
**The organism of CRBSI/1000 catheter days,** median (IQR)				
Gram-positive	13.3 (5.0–33.3)	12.8 (5.2–33.3)	13.3 (3.7–37.8)	0.877 ^b^
Gram-negative	16.6 (11.1–22.9)	12.6 (11.1–16.6)	19.9 (13.1–37.0)	0.022 ^b^
Fungal species	12.1 (7.5–18.1)	11.1 (4.1–14.0)	13.9 (11.1–25.3)	0.037 ^b^

TCS = taurolidine–citrate; CVC = central venous catheter; CRBSI = catheter-related bloodstream infection. ^a^ Pearson’s exact chi-square test. ^b^ Mann–Whitney U test.

**Table 3:** Effect of TCS usage on CRBSI rates.

CRBSI	RR	95% CI	*p*
Overall	0.78	0.70–0.87	<0.001
Gram-positive	0.78	0.65–0.93	0.006
Gram-negative	0.48	0.41–0.57	<0.001
Fungal species	0.63	0.52–0.77	<0.001

The adjusted RRs were derived from a Poisson regression model that included age, sex, diagnosis category, ostomy status, catheter type, and follow-up duration as covariates. Log(catheter-days) was used as the offset. No overdispersion was detected, and clustering could not be modeled due to low recurrence. All comparisons are between TCS and heparinized saline groups. TCS = taurolidine–citrate solution; CRBSI = catheter-related bloodstream infection; RR = rate ratio; CI = confidence interval.

## Data Availability

The datasets supporting the conclusions of this article are included within the article.

## Abbreviations

The following abbreviations are used in this manuscript:

IF	Intestinal failure
PN	Parenteral nutrition
IT	Intestinal transplantation
CVC	Central venous catheter
CRBSI	Catheter-related bloodstream infection
TCS	Taurolidine and citrate solution
IFALD	Intestinal failure-associated liver disease
CI	Confidence interval
RR	Rate ratio
